# Effect of self-etch primer application on the bond failure rate of a mandibular bonded lingual retainer

**DOI:** 10.1186/s12903-022-02691-4

**Published:** 2022-12-30

**Authors:** Fethiye Cakmak Ozlu, Sabahat Yazıcıoğlu

**Affiliations:** Department of Orthodontics, Faculty of Dentistry, University of Ondokuz Mayıs, 55270 Atakum, Samsun, Turkey

**Keywords:** Bond failure, Bonded lingual retainer, Self-etch primer (SEP), Relaps

## Abstract

**Background:**

The aim of this study was to examine the effect of self-etch primer (SEP) application on the bond failure rate of a mandibular bonded lingual retainer over 24 months.

**Methods:**

The average age of the 86 individuals included in this study was 17 years 4 months. After the removal of the orthodontic appliances, the lingual retainers, which were made of six-stranded stainless steel wire, were bent and bonded onto the lingual surface of all mandibular anterior teeth. The study was performed using a split-mouth design. In the study group, the SEP was administered to the teeth’s lingual surfaces. In the control group, they were etched using 37% phosphoric etchant liquid gel. After etching, the primer was applied. The adhesive resin was applied and the retainer was fitted. The patients were re-evaluated over 24 months. The first bond failures and the amount of adhesive remaining on the tooth were recorded as the adhesive remnant index (ARI) scores. The chi-square test was used to compare the bond failure rates (*P* = 0.231) and ARI scores between the groups (*P* = 0.162). The survival rates of the retainers were estimated using the Kaplan–Meier test (*P* = 0.237). The significance level was *P* < 0.05.

**Results:**

The bond failure rates, ARI scores, and survival rates did not differ significantly between the groups.

**Conclusions:**

The results of this study demonstrated that an SEP can be used successfully in mandibular lingual retainer bonding. In situations where saliva isolation is difficult, bonding a fixed lingual retainer with SEP is recommended.

## Objectives

Orthodontic relapse occurs with displacement of teeth after orthodontic treatment [1]. Orthodontic retention can be achieved with the use of removable retainers such as Hawley or vacuum-formed retainers [2]. Computer aided design/computer aided manufacturing (CAD/CAM) nitinol retainers, twisted stainless steel wire retainers, fibre-reinforced composite (FRC) retainers, titanium or gold chain retainers can be used as fixed retainers. In fixed or bonded retainers, adhesive and retainer wire are used together [3]. Their advantages include being independent of patient cooperation, allowing physiological movement of the bonded teeth, and being efficient and almost invisible [4, 5]. However, bond failures that occur through breaking at the wire–adhesive or adhesive–enamel interface are important disadvantages [6]. Bond failure is observed more frequently at the adhesive–enamel interface [7] and it occurs as a result of lack of moisture control and contamination of the enamel surface during bonding [8]. Surface contamination can occur in two critical stages: after the tooth surface has been etched and after the adhesive has been applied. Therefore, bonding may be compromised at these stages [9]. Self-etch primers (SEPs), which are effectively used in the bonding of orthodontic brackets [10], combine the etching and bonding steps. Previous studies have reported that SEPs perform well in both wet and dry environments and provide clinically acceptable bracket bonding after saliva contamination [11]. But, our search of the literature revealed no study on the effect of SEP use on failure rates in multi-strand wire lingual retainer bonding. The duration of success for the multi-strand wire was reported to be about 23 months [12]. Therefore, the aim in the present study was to examine the effect of SEP application on the bond failure rate of a mandibular bonded lingual retainer over 24 months. The null hypothesis of the study was that there is no difference between the bond failure rates of flexible mandibular lingual retainers bonded using SEP and 37% phosphoric etchant liquid gel with a primer.

## Materials and methods

The study was designed according to the modified CONSORT 2010 checklist and the registration date was 22/04/2022 (clinical trials.gov identifier: NCT05340595). The sample size of this prospective clinical study was calculated to be at least 26 individuals for 98.6% significance level and 95% reliability [13] and 86 participants were included in this study. Approval for this study was obtained from the Zonguldak Bülent Ecevit University Clinical Research Ethics Committee in Zonguldak, Turkey (2013/17).

Individuals with the following conditions were sought for inclusion in the study:

1. Individuals who will continue to the retention phase after fixed orthodontic treatment at the department of Orthodontics, University of Zonguldak Bülent Ecevit, Zonguldak/Turkey,
2. Presence of all mandibular incisor and canine teeth,
3. Good oral hygiene,
4. No caries,
5. No fractures,
6. Healthy periodontal condition,
7. No restorations,
8. No previous bonded retainer,
9. No traumatic parafunctional habits such as bruxism.

The average age of the 86 participants (72 female and 14 male) was 17 years 4 months (11–34 years). After the orthodontic appliances were removed, a mandibular alginate impression was taken, and a plaster study model was constructed. Retainer length was planned as 3–3 for 69 participants treated without extraction, and 5–5 for 17 participants treated with first premolar extraction. The lingual retainers, which were made of six-stranded stainless steel wire 0.0215 inches in diameter (American Orthodontics, Washington. Avenue, Sheboygan, USA), were bent on the study models. Technician's experience is important to obtain a retainer that is passive and fits the lingual surfaces of the involved teeth. Therefore, in this study, all retainers were fabricated by the same orthodontic technician.

**Fig. 1:**
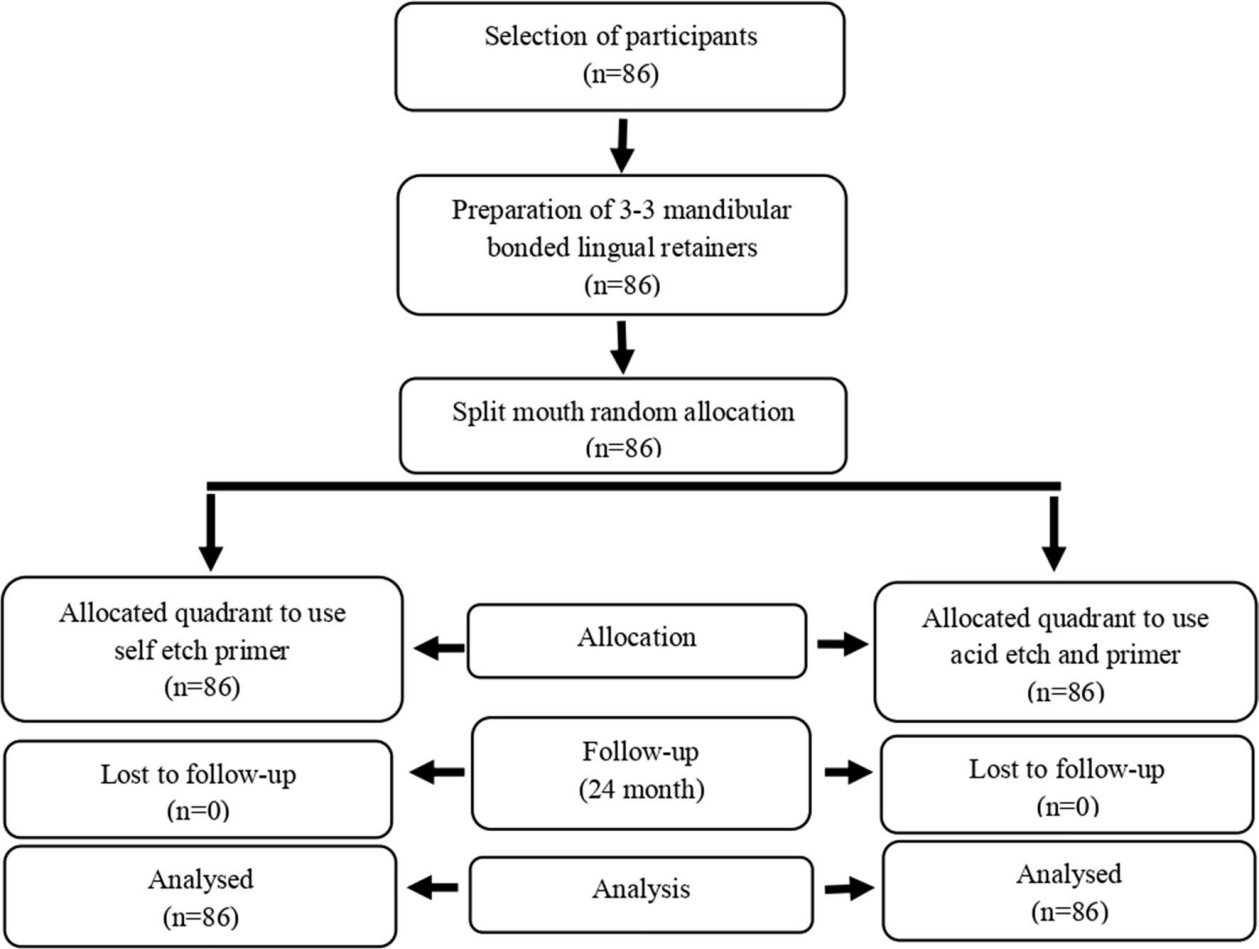
The Consort 2010 flow diagram of this study

To avoid inter-examiner variation, the bonding procedures were conducted by the same operator (FCO). Before bonding, non-fluoridated pumice was used for 20 s to polish the teeth. A split-mouth design was used. Thus, individual differences such as age, sex, enamel, and salivary structure and chewing function between the study and control groups were eliminated. The mouth was divided in half and a randomly alternating contralateral bonding pattern was used to make sure that the enamel treatment was equally distributed between right and left [14]. The direction of acid application was determined by drawing cards using a simple randomization method [15]. This randomization procedure was supervised by one operator (FCO). The Consort 2010 flow diagram of this study was shown in Fig. 1.

In the study group, the SEP (3 M Unitek, Monrovia, California) was used according to the manufacturer’s instructions, namely it was administered to the lingual surfaces of the teeth and rubbed for 3 s. Then a gentle burst of dry air was delivered to thin the primer. In the control group, the lingual surfaces of the teeth were etched using 37% phosphoric etchant liquid gel (3 M Espe, St Paul, Minnesota, USA) for 30 s, followed by rinsing and drying. Next, the primer (Transbond XT Primer; 3 M Unitek, California) was applied in a thin and uniform coat. Then the adhesive resin (Transbond LR Light Cure Adhesive Paste; 3 M Unitek, California) was administered to the lingual surface of the anterior teeth and the lingual retainer was placed. The adhesive resin was polymerized from two directions for a total of 20 s using a visible-light curing unit (Hilux 200, Benlioglu Dental Inc., Ankara, Turkey) with an output power of 600 mW/cm^2^.

The lingual retainer’s surface was examined for smoothness, the contact points and gingival areas for surplus adhesive.

The patients were evaluated again after 1, 3, 6, and 12 months. Then they were checked after 24 months. Bond failures were recorded by the same researcher (FCO). The amount of adhesive left on the tooth was ascertained visually using the adhesive remnant index (ARI) [16]. No drop-out occurred.

The statistical analysis was performed using the Statistical Package for the Social Sciences (version 12.0, SPSS Inc., Chicago, Illinois, USA). The bond failure rate over 24 months was established for both bonding procedures and only the first failures were used for the statistical analysis. The failure rates were compared using the chi-square test (*P* < 0.05). Chi-square analysis was also used to ascertain the differences in ARI scores between the bonding procedures (*P* < 0.05). The retainers’ survival rates were estimated by Kaplan–Meier test. Their survival distributions regarding the bonding procedure were compared using the log-rank test (*P* < 0.05).

## Results

The failure rates are given in Table 1. In the study group, bond failures occurred in 8 of the 86 retainers. The bond failure rate for the study group was therefore 9.3%. In the control group, they occurred in 4 of the 86 retainers. Thus, the bond failure rate for the control group was 4.7%. The bond failure rate did not differ significantly between the groups (*P* = 0.231).

**Table 1:** Bond failure rates

	No failure	Failure	Failure rate (%)	*P*
Study group	78	8	9.3	0.231
Control group	82	4	4.7	

χ^2^ = 1.433 on 1 degree of freedom (df)

The frequency distribution and the results of the chi-square analysis of the ARI scores are given in Table 2. Failures mostly occurred at the adhesive–enamel interface in the control group and at the adhesive–retainer interface in the study group. The bonding procedures did not differ significantly (*P* = 0.162).

**Table 2:** Frequency distribution and the result of the chi-square analysis of the adhesive remnant index (ARI)

	ARI scores			
	0	1	2	3
Study group	2	0	–	6
Control group	2	1	–	1

ARI scores: 0, no composite left on enamel surface; 1, less than half of composite left; 2, more than half of composite left; and 3, all composite left

χ^2^ = 3.643 on 2 df *P* = 0.162

**Fig. 2:**
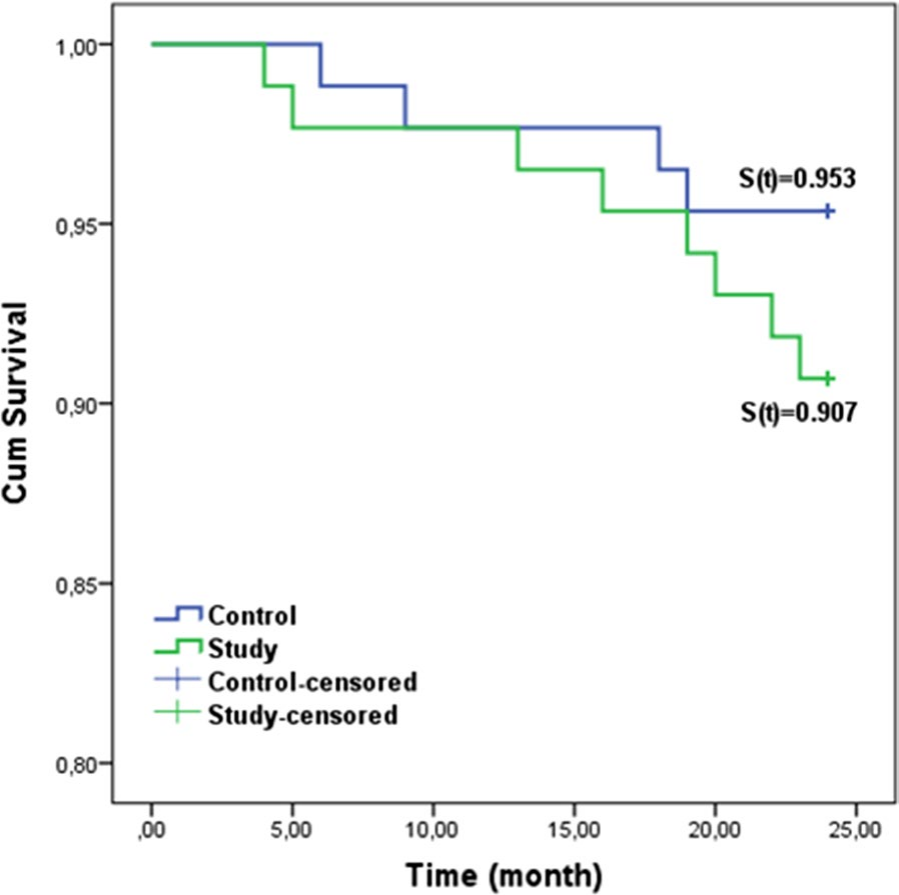
Retainer survival distribution for the bonding procedures

Survival curves were plotted using the Kaplan–Meier estimate and the effect of the bonding procedure on the retainer survival rate is presented in Fig. 2. According to the log-rank test there was no significant difference between the study (S[t] = 0.907) and control (S[t] = 0.953) groups (*P* = 0.237).

## Discussion

In the present study, the bonding procedure of lingual retainers, in individuals treated with or without extraction was evaluated. According to Little’s Irregularity Index, it was reported that crowding up to 3 mm: expansion, crowding between 3 and 5 mm: interproximal enamel reduction, and crowding above 5 mm: treatment with extraction may prevent the relaps [17]. However, the treatments with non-extraction or extraction of first premolars showed a similar tendency to incisor relapse [18]. The only way of ensuring good incisor alignment in the long term is permanent or semi-permanent retention. Nevertheless, the periodontal effects of permanent retantion definitely demand regular controls of plaque and periodontal treatment in susceptible patients  [19]. Since, prolonged retention may pose an increased risk to the periodontium and dental hard tissues [20, 21]**.** In addition, bonded retainers are likely to degrade the quality of diagnostic images and make diagnosis difficult. However, multi-stranded stainless steel retainers and titanium retainers cause minimal distortion in MRI when used in single or both arches. They are suitable materials for long time use as they do not need to be removed [22]. In this study, a six-stranded stainless steel retainer wire 0.0215 inches in diameter was attached to all anterior teeth. Retainers bonded to the anterior 6 teeth were reported to be more effective in preventing relapse [23]. A flexible multi-stranded wire bonded to each anterior tooth is considered the gold standard [24–26]

All retainers are bonded by the same clinician to eliminate individual practice differences in bonding. Jedliński et al. also emphasized the importance of the bonding skill of the clinician in the success of fixed retainers [27]. Bonding of lingual retainers by different clinicians affects failure rates [28].

In the present study, the enamel surface was cleaned by polishing with pumice before lingual retainer bonding. Keim et al. also suggested pumicing before lingual retainer bonding to minimize the risk of bond failure [29], because a clean and dry enamel surface is a critical factor affecting the success of retainer bonding [6].

In the control group of this study, the enamel surface was prepared by applying primer after 30 s of 37% phosphoric acid. In the literature, acid etch procedures used in fixed retainer bonding involve the application of phosphoric acid concentrations between 32 and 37% for between 15 and 60 s. The most common time reported was 30 s [27, 30].

Transbond LR which was used in the bonding of retainers made of 0.0125-inch six-stranded SS wire in previous studies, was used for bonding in this study [30–32] and Transbond XT Primer was used as the primer in the control group. It was reported that in the bonding of orthodontic retainers, Scotchbond Universal used in total-etch mode could be a valid alternative to the traditional orthodontic Transbond XT Primer [33]**.** It was reported that no primer adhesive GC Ortho Connect flow showed comparable shear bond strength (SBS) to Transbond XT in bonding of orthodontic retainers, but its higher microleakage may compromise its clinical success [34].

According to the results of the present study, the bond failure rate was 9.5% for the study group and 4.7% for the control group. Only the first failures were used for the statistical analysis [28]. However, these bond failure rates did not show a statistically significant difference between the two groups. In previous studies, failure rates for bonded retainers have been reported to range from 7.3 to 50% [27]. Additionally, the failure rates of six-stranded flexible spiral wire were less than 10% in the mandible up to 2–3 years [24, 35–37]. The bond failure rate in our SEP group was within this value range. Fleming et al. reported weak evidence that an SEP is more likely to fail than acid etch for full-arch bonded orthodontic appliances [7]. It has also been reported that SEPs provide clinically acceptable bond strength values when compared to acid etch after thermocycling [38]. Those researchers' results are for bracket bonding, but are important for comparing the effects of SEP and acid etch in bond failure.

In the current study, ARI scoring was used to assess whether bond failures occurred at the enamel–adhesive or adhesive–retainer interface. An ARI score of 3 is the type of failure that indicates the success of the adhesive–enamel bond, with all the adhesive left on the surface of the enamel. There were more ARI scores of 3 in the study group than in the control group. These values did not differ significantly between the groups. However, failures were mostly at the adhesive–retainer interface in the study group and at the adhesive–enamel interface in the control group.

Another factor to consider in bond failures in bonded lingual retainers is follow-up time [2]. The follow-up periods in randomized controlled trials (RCTs) and in case–control studies ranged from 6 months to 2 years [27]. Renkema et al. reported that the failures tend to occur mostly within 2 years after retainer placement [39]. Therefore, the follow-up period of this study was two years. During this period, the first bond failure observed for each tooth was recorded. Han et al. also recorded when during the follow-up the first breakage or loosening of the fixed retainer that occurred in their study [40]. Bond failures have been reported to occur mostly within the first 3–6 months of retention [28]. In this study, the bond failures occurred mostly in the first 6 months, but the survival rate did not differ significantly between the groups. The cumulative survival rate was 91% and 95% for the study and control groups, respectively, over an observation period of 24 months. Foek et al. [41] reported a total survival rate of 63% during their observation period of 41.7 months. Furthermore, Egli et al. [42] found a 60% survival rate for their 2-year observation period. The investigators' bonding methods were heterogeneous and applied by different clinicians. However, in our study, a standard bonding procedure was applied by a single operator. Therefore, it was thought that the survival rates for both groups were higher in this study than those in other studies.

In previous studies, serious and multiple bond failures for bonded mandibular retainers have been reported rarely [8]. In this study, the rate of multiple bond failure was also very low. Bonding with SEPs has been reported to have significantly shorter average bracket bonding time, i.e. chair time, than with acid etching [10]. The fact that the mean retainer bonding time with SEP was not calculated was regarded as a limitation of this study.

Last but not least, minimizing aerosol-generating procedures in orthodontics is of utmost importance during SARS-COV-2 pandemic like situations [43]. As mentioned above, SEP’ s shorten chair time. Thus, reduce aerosol-generation and exposure.

In future studies, the effect of SEP application in the repair of recurrent bond failures in bonded lingual retainers can be evaluated. In addition, SEP application can be compared with phosphoric acid concentrations lower than 37%.

## Conclusions

According to the results of this study, SEP application can be used successfully in mandibular lingual retainer bonding.

## Data Availability

The datasets used and/or analysed during the current study are available from the corresponding author on request.

## Abbreviations

%: Percent
ARI: Adhesive remnant index
SEP: Self-etch primer
s: Second
mW/cm^2^: MilliWatts per square centimeter
CAD/CAM: Computer aided design/computer aided manufacturing
FRC: Fibre-reinforced composite
SBS: Shear bond strength
